# Supplementary material for: Facile assembly of an affordable miniature multicolor fluorescence microscope made of 3D-printed parts enables detection of single cells
Source: PLoS One. 2019 Oct 10;14(10):e0215114. doi: 10.1371/journal.pone.0215114 (PMC6786622; doi:10.1371/journal.pone.0215114)
Supplement: S1 Table — (PDF) [file pone.0215114.s001.pdf]

Description	Manufacturer	PN	QTY	Cost each (USD)	Total
Mechanical assembly					
3D printed parts (10 pieces, ≈ 35 gr. PLA) *				2.5	2.5
		**Sub-total Mechanical**			***2.5***
Electronic components					
4N25 Optocupler	On Semiconductor	4N25SM	3	$0.60	1.8
BJT TIP-41	On Semiconductor	MJB41CG	3	$1.44	4.32
Voltage regulator LM317	Texas Instruments	LM317DCYR	3	$0.95	2.85
3 mm White LED	Lumex	SLX-LX3054UWC	1	$0.70	0.7
UV SMD LED, 385 nm	Vishay	VLMU3500-385-120	1	$11.46	11.46
High power Green LED, 535 nm	Multicomp	OSW-4334	1	$10.78	10.78
High power Blue LED, 475 nm	Multicomp	OSW-6303	1	$11.68	11.68
100 Ω SMD resistor	Yageo	RC0402JR-13100RL	3	$0.02	0.06
1 kΩ SMD resistor	Yageo	RC0402JR-131KL	3	$0.02	0.06
10 kΩ SMD resistor	Yageo	RC0402FR-1310KL	6	$0.02	0.12
2.2 Ω SMD resistor	Bourns	CRM2512-JW-2R2ELF	1	$0.19	0.19
3.3 Ω SMD resistor, 2512	Bourns	CRS2512-FW-3R30ELF	3	$0.44	1.32
Male pin header (40 pins)	MCM	PH1-40-UA	1	$0.47	0.47
FR-4 plain copper board*	Mextronics	TGN-16	1	$0.42	0.42
		**Sub-total Electronics**			***46.23***
Electrical					
Power supply charger 3.4 V - 12 V, 500 mA	Steren	ELI-055	1	9	9
		**Sub-total Electrical**			***9***
Optical					
UV filter (High-pass)	Roscolux	#3114 - THOUGH UV	1	0.19	0.19
Green Wratten filter (Band-pass)	Kodak	#58 Wratten filter	1	0.12	0.12
Red Filter (High-pass)	Roscolux	#19 - FIRE	1	0.19	0.19
CMOS Camera 8 Mpx	Raspberry Pi	RPI 8MP CAMERA BOARD	1	26.44	26.44
		**Sub-total Optical**			***26.94***
Computer system					
Single board computer	Raspberry Pi	Raspberry Pi 2 Model B	1	40	40
7" Touchscreen Display	Raspberry Pi	Touch display	1	78	78
		**Sub-total Computing**			***118***
				Total	***202.7***
