# Supplementary material for: Facile assembly of an affordable miniature multicolor fluorescence microscope made of 3D-printed parts enables detection of single cells
Source: PLoS One. 2019 Oct 10;14(10):e0215114. doi: 10.1371/journal.pone.0215114 (PMC6786622; doi:10.1371/journal.pone.0215114)
Supplement: S2 Table — (PDF) [file pone.0215114.s002.pdf]

S2 Table. Comparison of the number of pieces required to assembly different *DIY* microscopes. Only assembly elements (fabricated, reused or adapted) that render operational the optical system structure of the microscopes are considered. Each of these microscopes is capable to perform observations in both brightfield and fluorescence.

#	Microscope	# Pieces	Authors	Reference
1	100€ Microscope	32	Chagas et. al.	[17]
2	"Mini microscope"	11	Zhang et. al.	[16]
3	Automated microscope	9	Schaefer et. al.	[5]
4	ScanDrop Sensor	9	Golberg et. al.	[29]
5	***"BioARTS Microscope"***	***6***	***Tristan et. al.***	***This work***
