# Supplementary figures and images for: Facile assembly of an affordable miniature multicolor fluorescence microscope made of 3D-printed parts enables detection of single cells
Source: PLoS One. 2019 Oct 10;14(10):e0215114. doi: 10.1371/journal.pone.0215114 (PMC6786622; doi:10.1371/journal.pone.0215114)

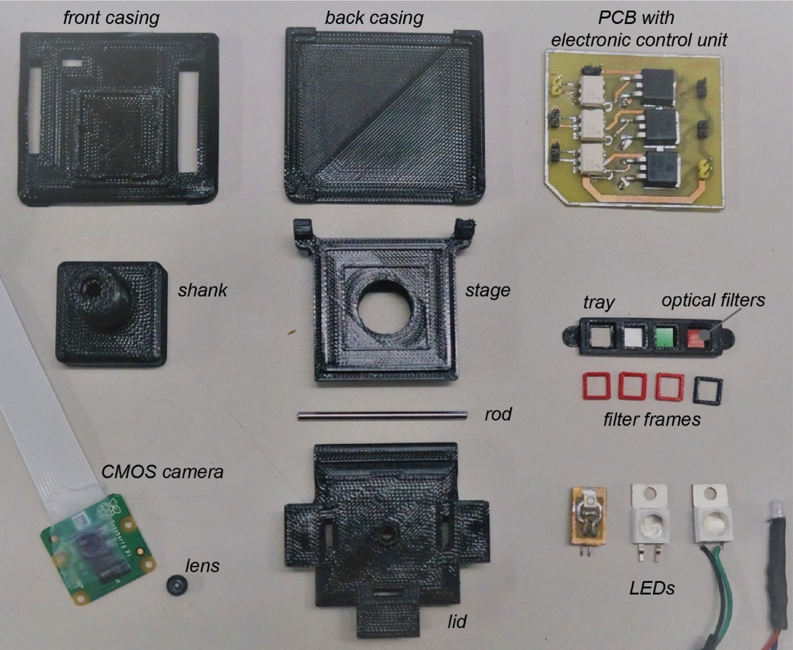

Supplement: S1 Fig — (TIF) [file pone.0215114.s007.tif]

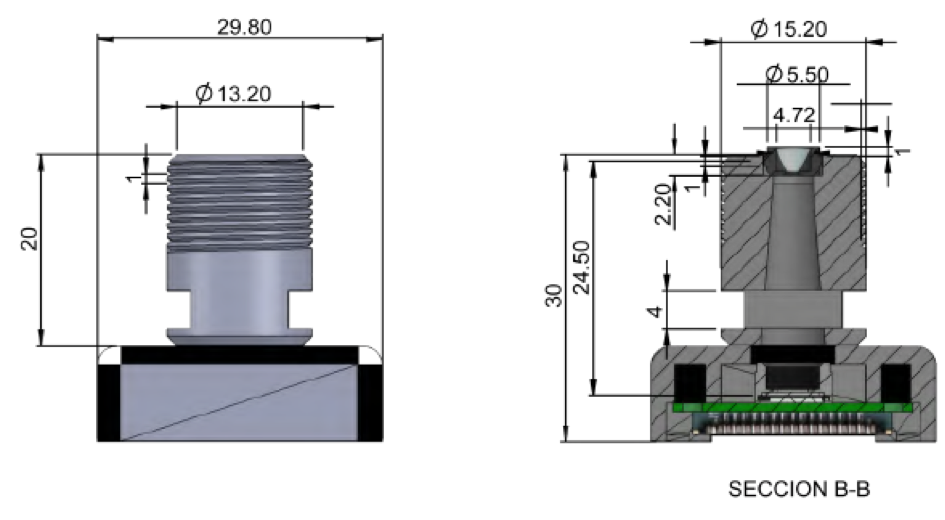

Supplement: S2 Fig — (Left) Front view of the mechanical tube of the microscope. Thread pitch is 1 mm. (Right) Cross-sectional view of the mechanical tube, showing the lens attached to the top of the tube. All dimensions are in mm. (TIF) [file pone.0215114.s008.tif]

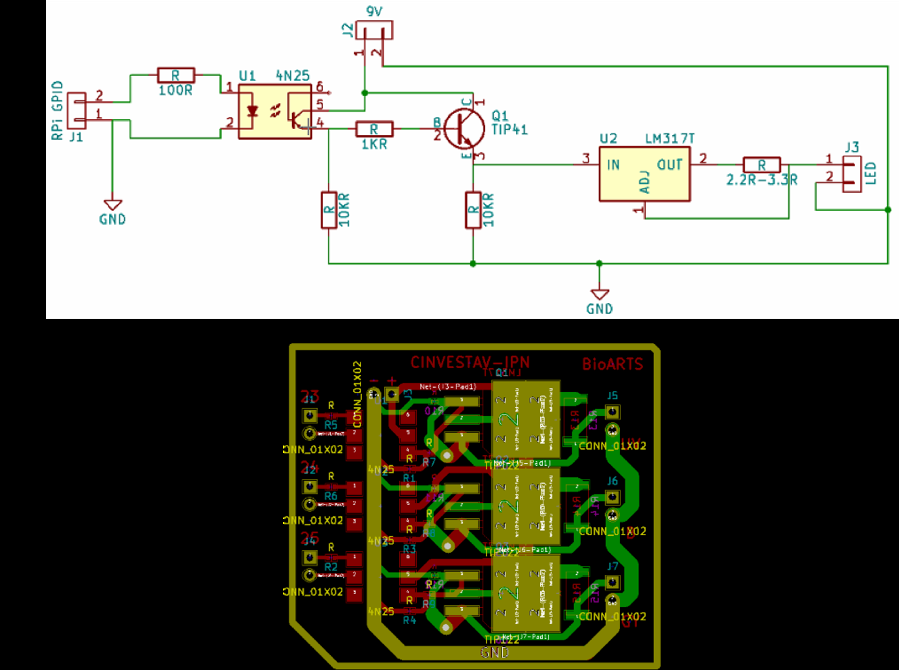

Supplement: S3 Fig — (a) Circuit design. (b) Printed circuit board layout. (TIF) [file pone.0215114.s009.tif]

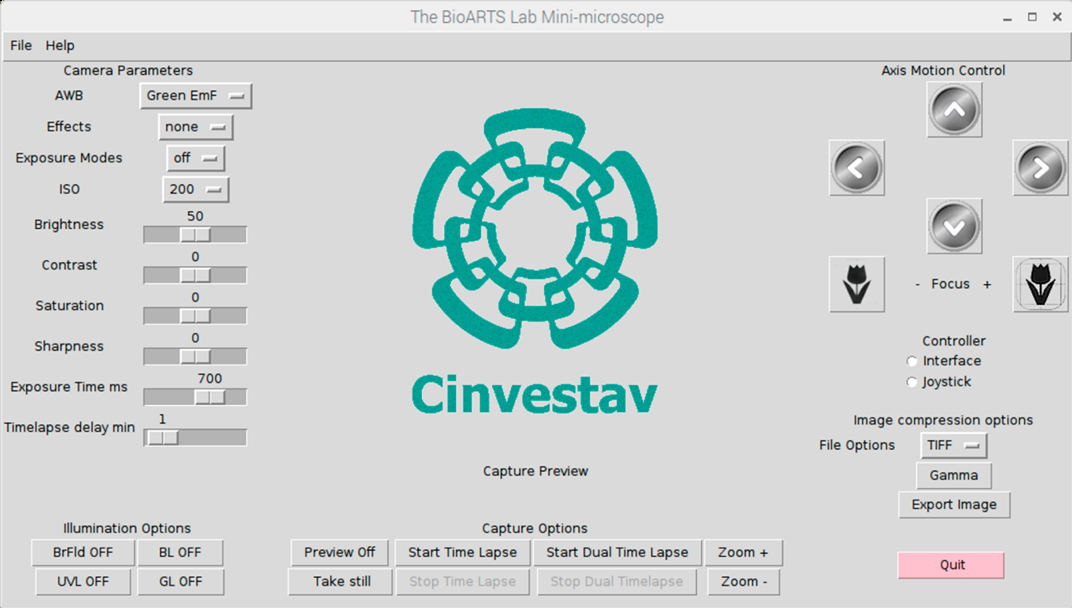

Supplement: S4 Fig — The GUI was coded in Python by combining Tkinter, picamera and OpenCV libraries. (TIF) [file pone.0215114.s010.tif]

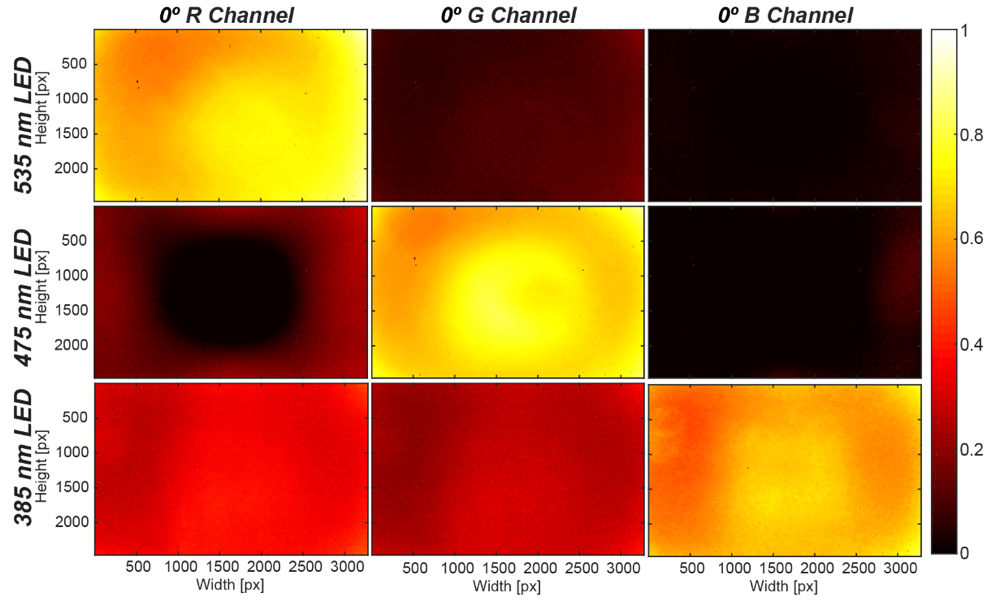

Supplement: S5 Fig — Each row corresponds to a different LED; the angle and the digital color channel analyzed are indicated on the top right corner. R: Red, G: Green, B: Blue. Fluorescence intensity has been normalized to a maximum value of 1. (TIF) [file pone.0215114.s011.tif]

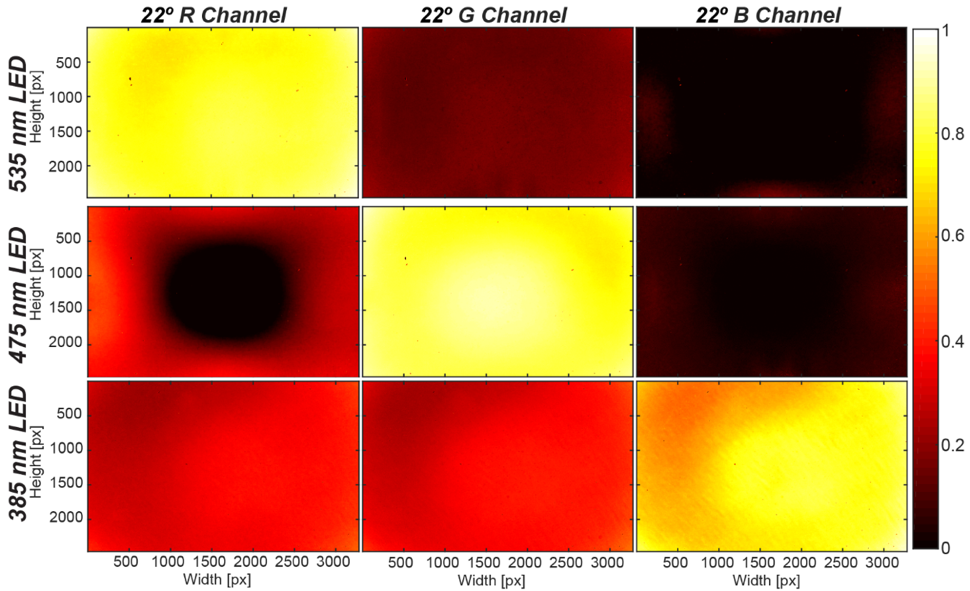

Supplement: S6 Fig — Each row corresponds to a different LED; the angle and the digital color channel analyzed are indicated on the top right corner. R: Red, G: Green, B: Blue. Fluorescence intensity has been normalized to a maximum value of 1 (TIF) [file pone.0215114.s012.tif]

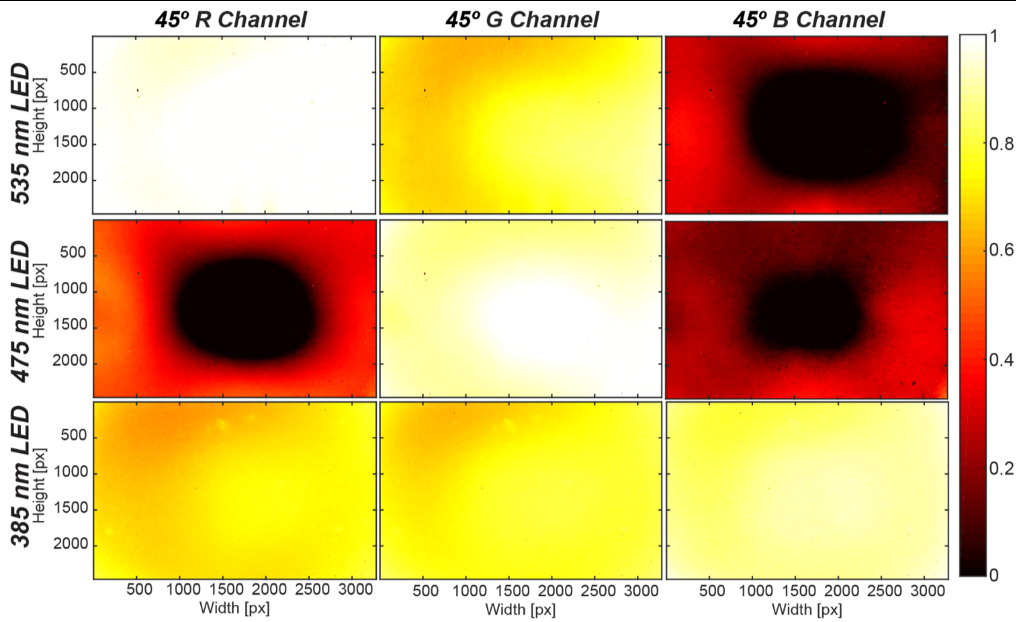

Supplement: S7 Fig — Each row corresponds to a different LED; the angle and the digital color channel analyzed are indicated on the top right corner. R: Red, G: Green, B: Blue. Fluorescence intensity has been normalized to a maximum value of 1 (TIF) [file pone.0215114.s013.tif]

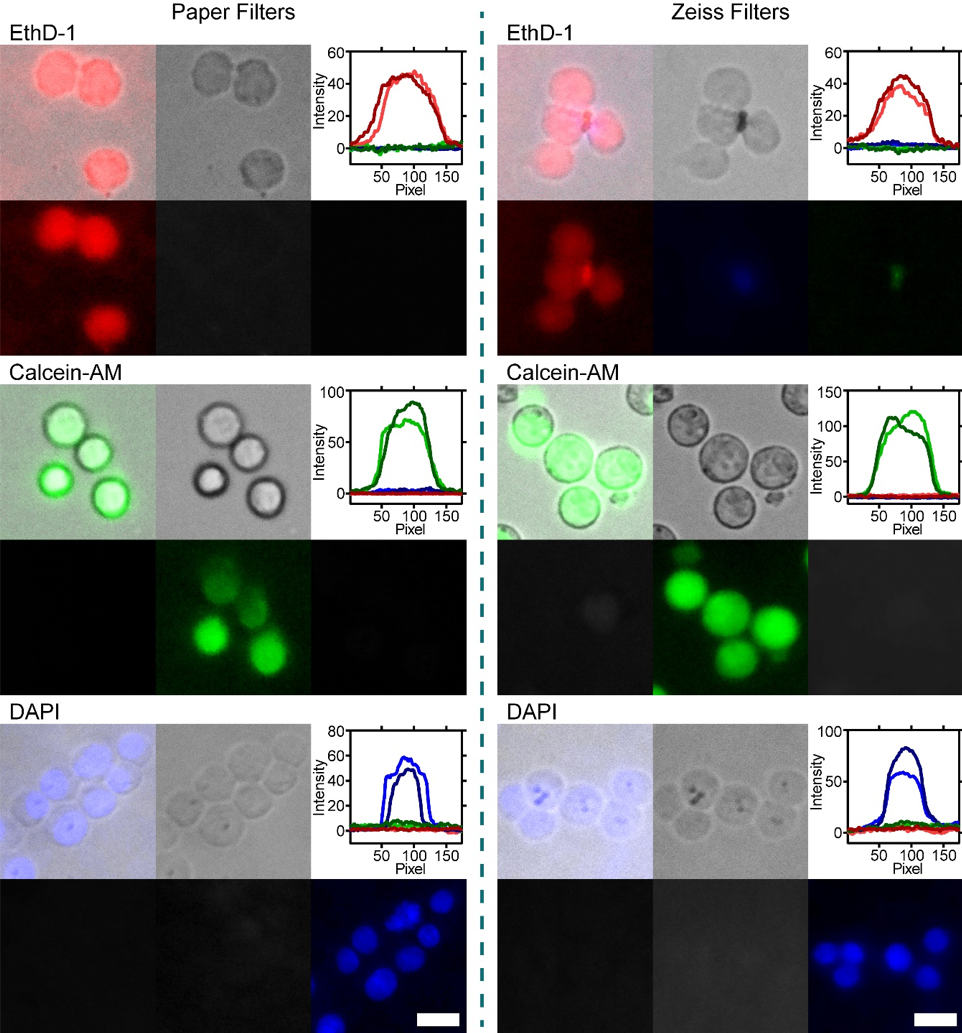

Supplement: S8 Fig — Bright-field and fluorescence micrographs captured with our microscope using plastic filters (left) or Zeiss filters (right). THP-1 cells stained with different fluorochromes: EthD-1 (red), Calcein-AM (green) and DAPI (blue). Images using all filters were acquired for each fluorochrome. Graphs show intensity profile of two cells across all channels, showing that there is no fluorescence bleed-through between channels. (TIF) [file pone.0215114.s014.tif]

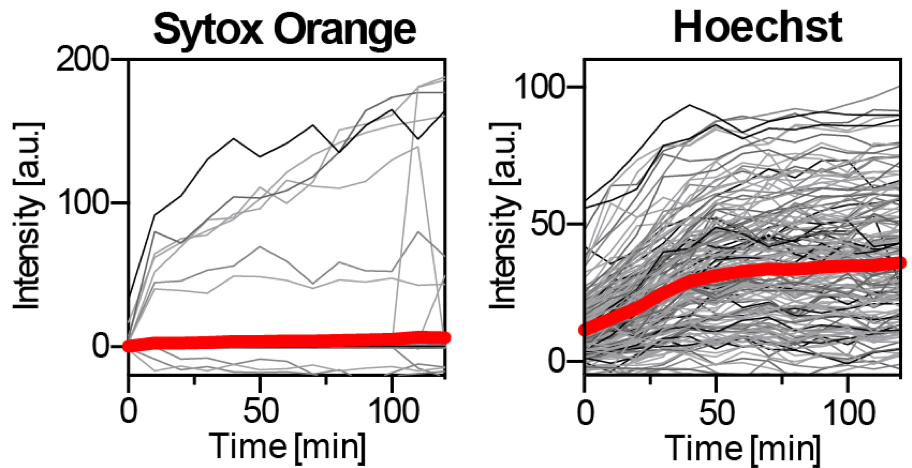

Supplement: S9 Fig — Traces of fluorescence intensities from single wells for the negative control experiment shown in Fig 6. (TIF) [file pone.0215114.s015.tif]

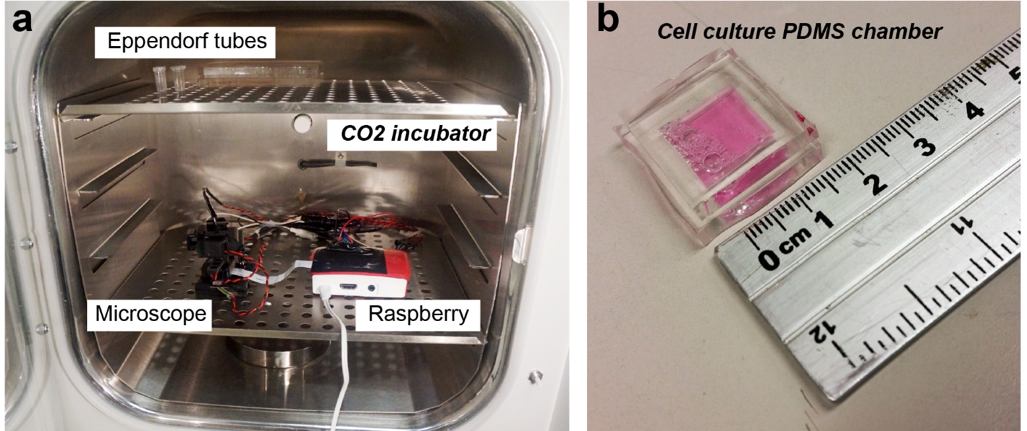

Supplement: S10 Fig — (a) Photograph of the inside of a cell culture incubator showing the microscope and the microcomputer Raspberry. (b) Photograph of the cell culture chamber made of PDMS. (TIF) [file pone.0215114.s016.tif]
